# Effect of miR-20b on Apoptosis, Differentiation, the BMP Signaling Pathway and Mitochondrial Function in the P19 Cell Model of Cardiac Differentiation In Vitro

**DOI:** 10.1371/journal.pone.0123519

**Published:** 2015-04-21

**Authors:** Shasha Zhu, Xiaoshan Hu, Zhangbin Yu, Yuzhu Peng, Jingai Zhu, Xuehua Liu, Mengmeng Li, Shuping Han, Chun Zhu

**Affiliations:** 1 Department of Cardiology, The First Affiliated Hospital of Soochow University, Suzhou 215006, China; 2 State key Laboratory of Reproductive Medicine, Department of Pediatrics, Nanjing Maternity and Child Health Care Hospital Affiliated to Nanjing Medical University, Nanjing 210004, China; Toho University School of Medicine, JAPAN

## Abstract

**Objective:**

To explore the effect of miR-20b on apoptosis, differentiation, the BMP signaling pathway and mitochondrial function in the P19 cell model of cardiac differentiation in vitro.

**Methods:**

A miR-20b over-expression vector, a miR-20b silencing vector and their corresponding empty vectors were constructed and transfected into P19 cells, separately. Stably miR-20b overexpressing and silenced P19 cell lines were successfully selected by blasticidin and puromycin, separately. The cells were induced to undergo apoptosis in FBS-free-α-MEM. The induced cells were examined by flow cytometry and measurement of their caspase-3 activities. Quantitative real-time reverse transcription polymerase chain reaction (qRT-PCR) was used to evaluate the relative expression of marker genes of cardiomyocytes during differentiation, such as cTnT, GATA4 and ANP. QRT-PCR was also used to detect the mitochondrial DNA (mtDNA) copy number. We investigated the cellular ATP production using a luciferase-based luminescence assay. The reactive oxygen species (ROS) was determined by DCFDA (2’, 7’-Dichlorofluorescein diacetate) and the mitochondrial membrane potential (MMP) was elucidated by a JC-1 fluorescent probe, both using fluorescence microscopy and flow cytometer. The expression of BMP signaling pathway-related proteins were analyzed by Western blotting.

**Results:**

Stably miR-20b overexpressing and silenced P19 cell lines were successfully obtained. MiR-20b overexpression increased apoptosis and promoted differentiation in P19 cells by promoting the activation of the BMP signaling pathway. In addition, miR-20b overexpression induced mitochondrial impairment in P19 cells during differentiation, which was characterized by lower MMP, raised ATP synthesis and increased ROS levels. The effects of miR-20b silencing were the exact opposite to those of overexpression.

**Conclusion:**

Collectively, these results suggested that miR-20b was very important in apoptosis, differentiation and mitochondrial function of P19 cells. MiR-20b may represent a new therapeutic target for congenital heart diseases and provide new insights into the mechanisms of cardiac diseases.

## Introduction

The vertebrate heart is the first functional organ to form during embryonic development, and is derived from the mesodermal cells that are enriched cardiomyocytes and endocardial cells in early embryos [1]. The formation of a mature healthy heart, having four chambers, relies on the sequential expression of many genes, a variety of signaling pathways, such as the BMP signaling pathway, the Wnt signaling pathway, and a series of important morphological changes, including cell migration and differentiation [2,3]. Many studies have shown that cardiac malformation occurs if mutations or deletions exist in any part of the above procedures [4–6], with a prevalence of approximately eight in every 1000 newborn infants [7], which places a heavy burden on families and society.

Congenital heart diseases (CHDs), which account for about 40% of perinatal deaths and more than one fifth of deaths in the first month of life, have been studied intensively by the international community and much progress has been made in recent years; however, the molecular mechanisms remain unclear [8]. The majority of CHDs are related to gene deletions and mutations [9,10]. Thus, genetic studies are the key to the prevention and treatment of CHDs.

MicroRNAs (miRNAs), a 19-23nt non-coding small RNA, are recently discovered to be active in CHDs. To date, more than 800 miRNAs have been identified in animals, and involved in cell proliferation, apoptosis, growth and differentiation [11–13]. The mature miRNA is incorporated into an RNA-induced silencing complex (RISC) by binding to a target messenger RNA (mRNA), to suppress or reduce the expression of post-translational protein [14,15]. An association between miRNAs and cardiogenesis and heart diseases has been confirmed [16,17]. For example, knockout of miRNA-1-2 led mice to develop a ventricular septal defect, pericardial edema and even death at embryonic day 15.5 (E15.5) [4]. MiR-423-5p, miR-208a and miR-1 all played important roles in acute myocardial infarction and stable coronary heart disease [18,19]; the abnormal cardiomyocyte development and ventricular dysfunction in zebrafish happened because of the lack of miR-138 [5], etc.

**Fig 1:**
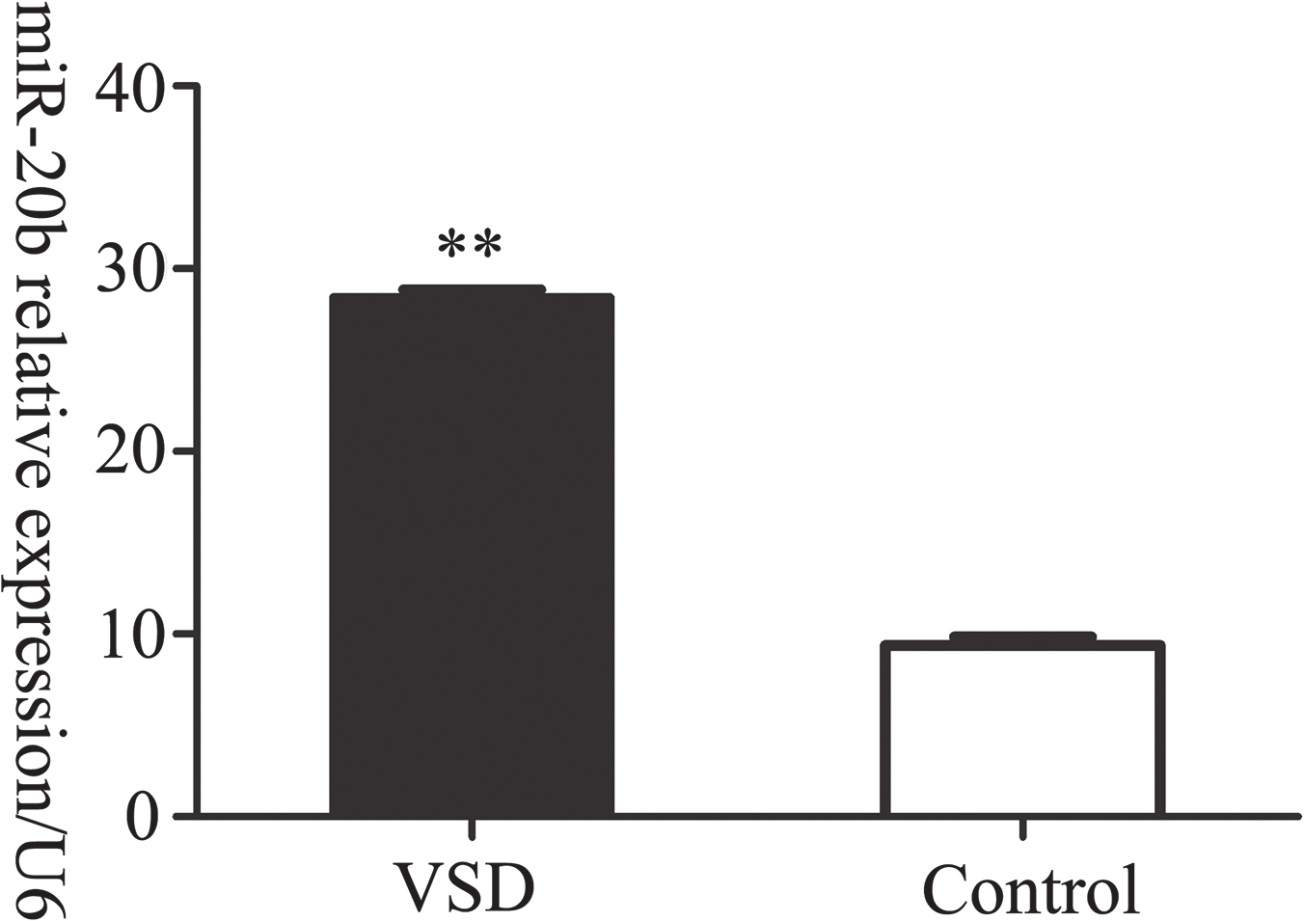
The relative expression of miR-20b in ventricular septal defect. VSD: ventricular septal defect group; control: healthy group.

In our previous trials, the highly conserved miR-20b was found to be differentially expressed in the aborted embryonic heart tissues of ventricular septal defect (VSD) in the second trimester using a microarray. We have validated the results by real-time PCR (Fig 1). Mmu-mir-20b, a member of the miR-17 microRNA precursor family, is expressed in embryonic hearts of many organisms, such as zebrafish, rat and mouse, etc [20,21]. Therefore, we hypothesized that miR-20b may be associated with cardiogenesis. Mir-20b could induce B7-H1 gene overexpression by inhibiting PTEN in advanced colorectal cancer [22]. In MCF-7 breast cancer cells, miR-20b regulated vascular endothelial growth factor (VEGF) by acting on HIF-1α and STAT3 [23]. In addition, the distribution of miR-20b affected breast cancer metastasis and heterogeneity [24]. Although, miR-20b is involved in the pathogenesis and development of a variety of cancers, the mechanism of miR-20b’s involvement in cardiogenic processes remains poorly understood.

Bone morphogenetic proteins (BMPs), members of the transforming growth factor –β (TGF-β) superfamily, contain a highly homologous conserved domain and play critical roles in cardiac cell differentiation [25–28]. It has been shown that the BMP signaling pathway promoted P19CL6 (a cloning derivative of P19 cell) differentiation into cardiac cells. In addition, overexpression of BMP could reverse the failure of P19-deficient cells differentiation [26]. BMPs combined with Alk3 and BMPR2 to regulate the expression of GATA4 and Nkx2.5, ultimately resulting in cardiac differentiation [27,28]. BMP and activin membrane-bound inhibitor (Bambi) is a highly evolutionarily conserved transmembrane glycoprotein. Its structure is similar to BMP receptors; therefore, it is considered as a pseudo-receptor of BMP that can also bind with BMPs to form aggregates. Bambi lacks the intracellular kinase fragment; therefore, the downstream proteins cannot be phosphorylated to improve the activity of the BMP signaling pathway [29–31]. Bioinformatics analysis showed that Bambi was a potential target gene of mmu-miR-20b. Thus, we presumed that the effect of miR-20b on Bambi might alter the activity of the BMP signaling pathway and be associated with cardiac malformations.

P19 cells, isolated from an experimental embryo-derived mouse teratocarcinoma, are pluripotent stem cells cultured in vitro, which have the ability to self-replicate and differentiate [32,33]. P19 cells, which can differentiate into cardiomyocytes at low concentrations of dimethyl sulfoxide (DMSO), are used widely in cardiogenesis as a myocardial cell model [34].

In this study, we explored the effects of miR-20b on apoptosis, differentiation, BMP signaling pathway and mitochondrial function in the P19 cell model of cardiac differentiation in vitro. We analyzed the molecular mechanisms of miR-20b during myocardial differentiation to provide new clues and a theoretical basis for the cause of cardiac malformations, and to discover new targets for the early prevention and treatment interventions of CHD.

## Materials and Methods

### Cell Culture and Induction of Differentiation

The mouse embryonic carcinoma (P19) cells used in this study were obtained from the American Type Culture Collection (ATCC, Manassas, VA, USA). The cells were maintained in α-minimal essential medium (α-MEM, Gibco BRL, Grand Island, NY, USA) supplemented with 10% fetal bovine serum (FBS, Gibco BRL), 100U/ml penicillin, and 100ug/ml streptomycin at 37°C in a 5% CO_2_ atmosphere. To induce cardiac differentiation, P19 cells were cultured as aggregates for 4 days in the presence of α-MEM containing 10% FBS and 1% dimethyl sulfoxide (DMSO, Sigma, St. Louis, MO, USA) on 10-cm bacterial dishes at 37°C in an atmosphere of 5% CO_2_ in air. The medium was replaced every 2 days. The embryoid bodies (EBs) were transferred to 6-well cell culture plates on day 4. The medium was replaced with α-MEM only supplemented with 10% FBS for an additional 6 days. We harvested the cells at differentiation day 0, 4, 6, 8 and 10. The morphological changes during the differentiation of P19 cells into cardiomyocytes were observed under an inverted microscope (Nikon ECLIPSE TP300, Tokyo, Japan) equipped with phase-contrast objectives and a digital camera (Nikon E4500). To examine whether P19 cells differentiated into cardiomyocytes, we identified the expression of cardiac troponin T (cTnT) by western blotting using a monoclonal rabbit anti-cTnT antibody (SANTA, USA) during differentiation, which would be described later.

### Construction of Expression Plasmids

The mature sequence of mouse miR-20b (mmu-miR-20b) was amplified and cloned into the vector pPG/miR/eGFP/Blasticidin (GenePharma, Shanghai, China) using EcoRI and AgeI as the specific restriction enzyme to construct a miR-20b overexpression vector. The mature sequence of mmu-miR-20b was: CAAAGTGCTCATAGTGCAGGTAG. The sense strand was: (5′-3′) ACC**AATTC**GCAAAGTGCTCATAGTGCAGGTAGGTTTTGGCCACTGACTGACCTACCTGCACTGAGCACTTTGCA; and the antisense strand was: (5′-3′) GCC**CCGGT**GCAAAGTGCTCAGTGCAGGTAGGTCAGTCAGTGGCCAAAACCTACCTGCACTATGAGCACTTTGCG (restriction enzyme sites are shown in bold face).

We used Targetscan software to analyze the potential miRNA target sites in the 3′UTR of mouse *Bambi*. We inserted the fragment including the 3′UTR (or mutant 3′UTR) regions of Bambi into XhoI/NotI-digested vector psiCHECK-2 (GenePharma, Shanghai, China) containing a firefly and renilla luciferase reporter gene. The mutant sites were shown in boldface in the following sequences: 3′UTR region of *Bambi*: GTTCTGCTGACAGGAGCACTTTT; mutant 3′UTR region of Bambi: GTTCTGCTGACAGGA**CGTGAAA**T.

In the study, we used a novel miRNA interference technique, “miRNA sponge”, to suppress endogenous expression of miR-20b. The miRNA sponge silenced miR-20b at the presence of a plurality of complementary antisense sequences. A fragment, containing four antisense sequences of mmu-miR-20b-5p, was linked to BamHI/EcoRI-digested vector pGLV3/H1/GFP/Puro (GenePharma, Shanghai, China). The antisense sequence of mmu-miR-20b-5p was CTACCTGCACTATGAGCACTTTG.

### MiRNA Transfection and Establishment of Stable Cell Lines

The overexpression vector, the silencing vector and their corresponding empty vectors were transfected into P19 cells by Lipofectamine-2000 (Invitrogen, USA) when the cells were about 50% confluent. The stable cell lines overexpressing miR-20b were screened with 60μg/mL Blasticidin S HCl (Invitrogen, Carlsbad, CA, USA) and maintained in culture with 300μg/mL of Blasticidin. Cells stably maintaining the silencing construct were selected on 2μg/mL puromycin (Invitrogen, Carlsbad, CA, USA) and maintained in culture with 1μg/mL of puromycin. The establishment of miR-20b overexpressing cells and miR-20b-silenced cells were confirmed by qRT-PCR and luciferase assay, respectively (see below).

### Flow Cytometry and Measurement of Caspase-3 Activity: Apoptosis Assay

Cells were cultivated in serum-free α-MEM for 24h to induce apoptosis, and then harvested by trypsin/EDTA (Gibco BRL, USA) and washed with phosphate-buffered saline (PBS, Gibco BRL). The cells were centrifuged and resuspended in 1mL binding buffer, and stained with 5μL Annexin V-APC and 5μL 7-AAD at room temperature for 15 min (Biovision, CA, USA). Flow cytometry was then used to analyze the cells immediately.

A Caspase 3 Activity Detection Kit (Biovision, CA, USA) was used to analyze the cells in which apoptosis had been induced, according to the manufacturer’s instructions. An ELISA reader measured the absorbance values at 400–405nm. The difference among these values was used as the optical density (OD).

Quantitative Real-Time Reverse Transcription Polymerase Chain Reaction (qRT-PCR) Total RNA, including miRNAs, was exacted from miR-20b overexpressing cells and their controls using a miRVana PARIS Kit (Ambion, Foster City, USA), according to the manufacturer's protocols. RNA was detected by a NanoDrop2000 Spectrophotometre (NanoDrop Tech., Rockland, De, USA) to assess its quality and quantity, and stored at -80°C. A Taqman MicroRNA Reverse Transcription Kit and miRNA-specific stem-loop primers (Life Tech., USA) on a PCR system (Bio-Rad, USA) were then used to reverse transcribe the total RNA into cDNA. We used U6 as an internal reference. Real-time PCR (Taqman method) was conducted in 96-well plates on the ABI 7500 Real-time PCR system (Applied Biosystems, USA). The primer IDs of miR-20b and U6 were shown in Table 1.

**Table 1:** Taqman assay IDs of genes used in Taqman qPCR.

Gene	Taqman Assay ID
mmu-miR-20b	00104
U6	001973
cTnT	Mm01290256_m
ANP	Mm01255747_g1
GATA4	Mm00484689_m1
β-actin	Mm00607939_s1

The TRIzol reagent (Invitrogen, USA) was used to isolate total RNA, whose concentration was measured as above. A High Capacity cDNA Reverse Transcription Kit (Life Tech.) was then used to synthesize cDNA. Real-time PCR (Taqman method) were initiated at 95°C for 10min, followed by 40 cycles of 95°C for 15s and 60°C for 1min. The Taqman Assay IDs were also shown in Table 1. β-actin was chosen as an internal control to measure the relative expression of cardiac marked genes (cTnT, ANP, GATA4), which was determined with the comparative cycle threshold (CT) (2^-ΔCT^) method, in which ΔCT = C_T genes_ - C_T β-actin_.

Real-time PCR was used to detect the mitochondrial DNA (mtDNA) copy number, as described previously [35]. We used a DNA Exaction Kit (QIAGEN, Dusseldorf, Germany) to exact DNA from differentiated cells. Two primer sets (listed in Table 2) were used for PCR analysis (SYBR Green method). A 110-nt mtDNA fragment within the cytochrome B gene (CYTB) was used to quantify mtDNA. Previously, we inserted the PCR product into plasmid pMD-T18, which was confirmed by DNA sequencing. A log-linear standard curve was generated from the plasmid standards of known copy number, through which the CYTB copy numbers of the samples could be determined by real-time PCR conducted on the ABI 7500 Sequence Detection System. We used a 291-bp region of nuclear gene for 28S as a normalization standard. The copy number of mitochondrial per cell was reflected by the ratio of mtDNA to nuclear DNA.

**Table 2:** Sequences of primer used in SYBR Green qPCR.

Gene	Forward primer(5'-3')	Reserve primer(3'-5')
CYTB	TTTTATCTGCATCTGAGTTTAATCCTGT	CCACTTCATCTTACCATTTATTATCGC
28S	GGCGGCCAAGCGTTCATAG	AGGCGTTCAGTCATAATCCCACAG

### Luciferase Assay

The psiCHECK-2 vectors, containing 3′UTR regions or mutant 3′UTR regions of Bambi, were transfected into miR-20b overexpressing and miR-20b silenced cells, and their controls, separately. Twenty-four hours later, the Dual Luciferase Reporter Assay System (Promega, USA) was used to measure the firefly and renilla luciferase activities according to the manufacturer's protocols.

### Antibodies and Western Blotting

Anti-cTnT and anti-β-actin antibodies both purchased from Santa Cruz Biotechnology (Santa Cruz, CA, USA), anti-GATA4 and anti-Nkx 2.5 antibodies were both from Affinity BioReagents (Affinity, USA). Anti-Bambi and anti-Alk3 antibodies were bought from Proteintech Group (Proteintech, USA). All these antibodies were monoclonal rabbit antibodies. The lysis buffer provided in the Total Protein Extraction Kit (KeyGen, inc., China) was used to break the differentiated cells. We obtained the lysate supernatant after centrifugation at 14000×g for 30min at 4°C. Subsequently, the concentration of the protein was analyzed by a BCA Protein Detection Kit (KeyGen) according the manufacturer’s protocol. Western blotting was performed as previously described [36].

### Measurement of Cellular ATP Production

A Luciferase-Based Luminescence Assay Kit (Biyutian, Nantong, China) measured cellular adenosine triphosphate (ATP) on the 10^th^ day of differentiation. Briefly, we homogenized the stable P19 cells in an ice-cold ATP-releasing buffer, 20μl of which was assayed in a single-tube luminometer (Turner, Biosystems, CA) together with 100μl ATP detection buffer. A standard curve of ATP concentration (1 nM–1μM) was drawn from a known amount of ATP. The production of ATP was normalized to protein concentration.

### Fluorescence microscopy and flow cytometry: Assessment of Intracellular Oxidation Level and Mitochondrial Membrane Potential (MMP)

The generation of ROS was measured by a DCFDA probe (Sigma, St. Louis, MO, USA) [37], while MMP was assessed using a JC-1 fluorescent probe from the Mitochondrial Membrane Potential Detection Kit (Biyutian, China). The cells, washed twice with phosphate-buffered saline (PBS) buffer, were incubated in 1ml of JC-1 and 5μM of DCFDA for 30min at 37°C to stained, washed three times with pre-warmed PBS again, and then viewed under a fluorescence microscopy, respectively. For flow cytometry, following trypsinization and centrifugation, the stained cells were washed and resuspended cells in 300μl PBS buffer. We analyzed the fluorescence with a FACScan flow cytometer with the CellQuest software (BD Biosciences, San Joes, CA, USA).

### Statistical Analysis

All statistical analysis was performed using the Student’s t-test or one-way analysis of variance (ANOVA) with SPSS software version 13.0 (SPSS, Inc, Chicago, USA). P-values less than 0.05 were considered statistically significant, and all the data were expressed as the mean ± the standard deviation (SD) from at least three independent experiments.

## Results

### Validation of stable miR-20b overexpression and silencing in P19 cells and appraisal of the target gene (Bambi)

**Fig 2:**
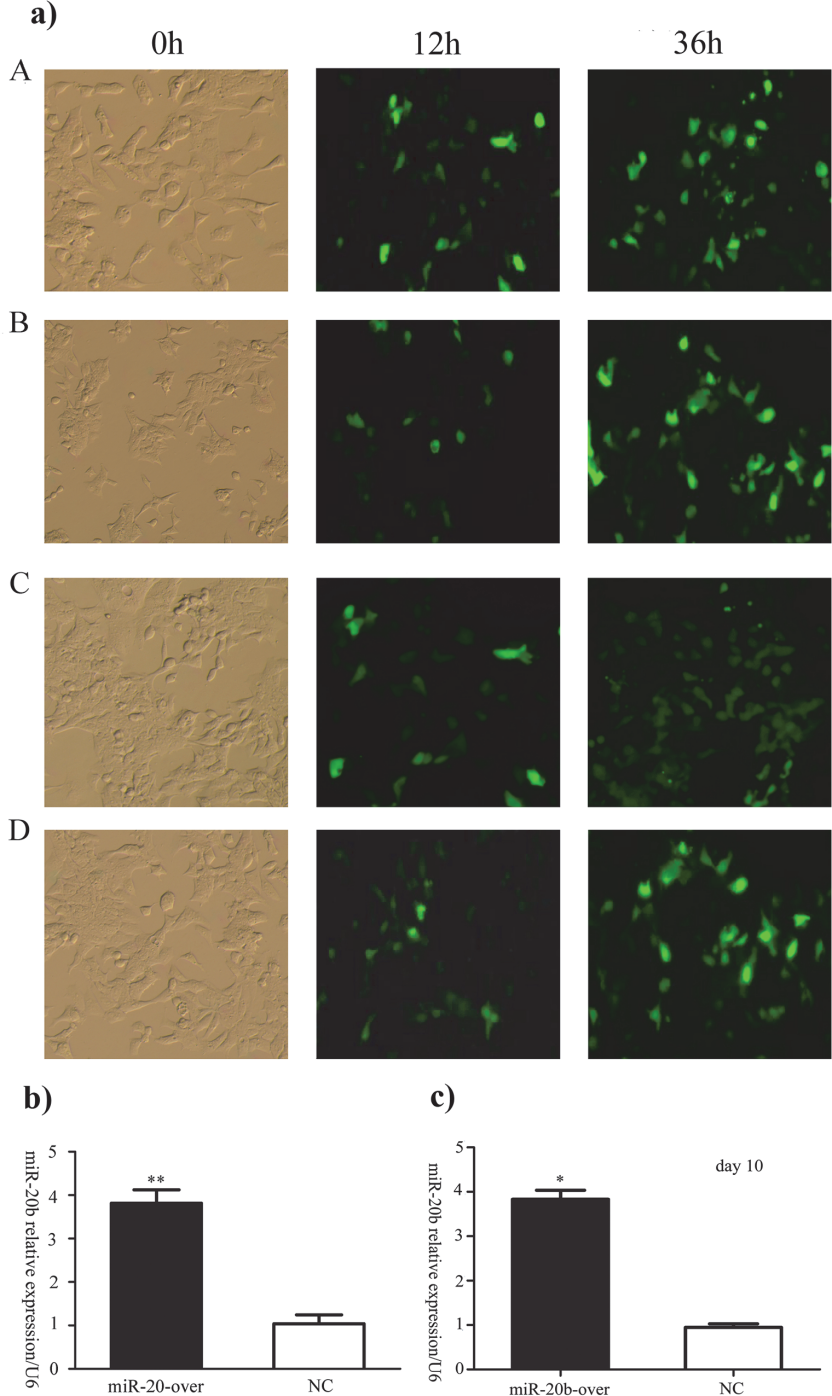
The transfection efficiency in P19 cells was similar (a). A: control for miR-20b overexpression. B: miR-20b overexpression. C: control for miR-20b silencing. D: miR-20b silencing. **miR-20b overexpression confirmed by qPCR. c) miR-20b overexpression constant during the differentiation (day 10) by qPCR (b).** (n = 6, **: P<0.01, *: P<0.5)

After the overexpression vector, the silencing vector of mmu-miR-20b and their corresponding empty vectors were transfected into P19 cells, respectively, the GFP expression was observed under a fluorescence microscope. The results indicated that the transfection efficiencies were similar (Fig 2A). As shown in Fig 2B and Fig 2 coverexpression of miR-20b was confirmed by qPCR and constant during the differentiation condition (day 10) (**: P<0.01, *: P<0.5).

**Fig 3:**
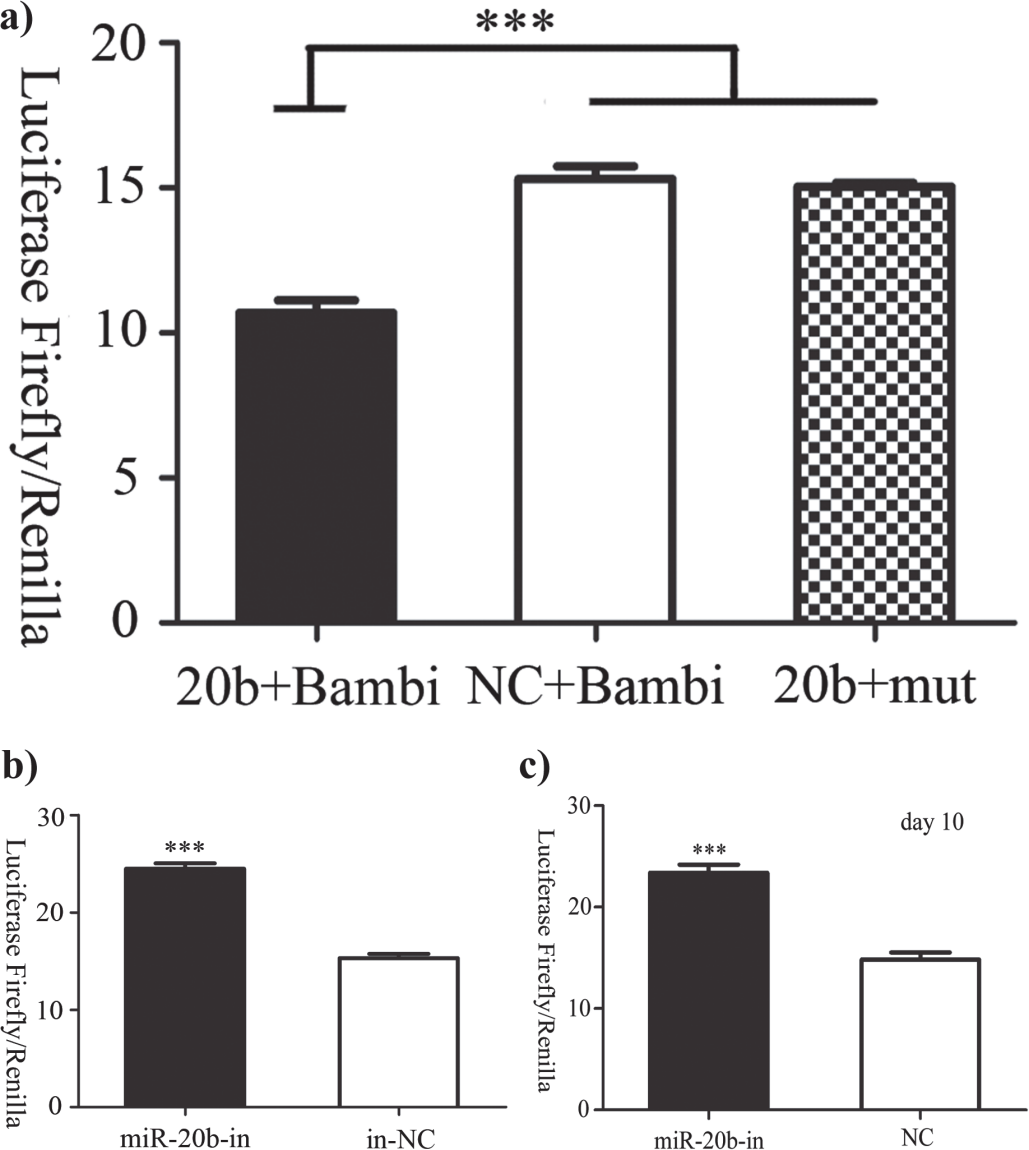
Luciferase activity assessed by the Dual Luciferase Reporter Assay System. **a) Bambi confirmed as the direct target gene of miR-20b.** 20b+Bambi: the luciferase activity of Bambi in the miR-20b overexpression group; NC+Bambi: the luciferase activity of Bambi in the control; 20b+mut: the luciferase activity of mutated Bambi in the miR-20b overexpression group. **b) miR-20b silencing confirmed. c) miR-20b silencing constant during the differentiation (day 10)** (n = 6, ***: P<0.001)

We identified Bambi, which is involved in cell differentiation, as a potential target gene of miR-20b by a variety of online gene analysis software (Targetscan 5.1, miRanda and DIANA-microT 3.0). Dual Luciferase Reporter, a quick and efficient tool, detected the expression of Bambi in response the various miRNA constructs. When miR-20b was bound with the target site, the luciferase activity of Bambi was inhibited. As results, in the miR-20b overexpression group, the luciferase activity of the psiCHECK-2-Bambi-3′UTR reporter was significantly lowered, whereas that of the mutated construct was not (mut- psiCHECK-2-Bambi-3′UTR), which suggested that Bambi was indeed the direct target gene of miR-20b (Fig 3A, P<0.001). In the miR-20b silenced cells, the luciferase activity of Bambi was significantly higher than the control, which suggested that a stable miR-20b silencing vector was established successfully (Fig 3B, ***: P<0.001). The silencing was constant during the differentiation condition (day 10) (Fig 3C, ***: P<0.001).

### Differentiation of P19 cells cardiomyocytes

**Fig 4:**
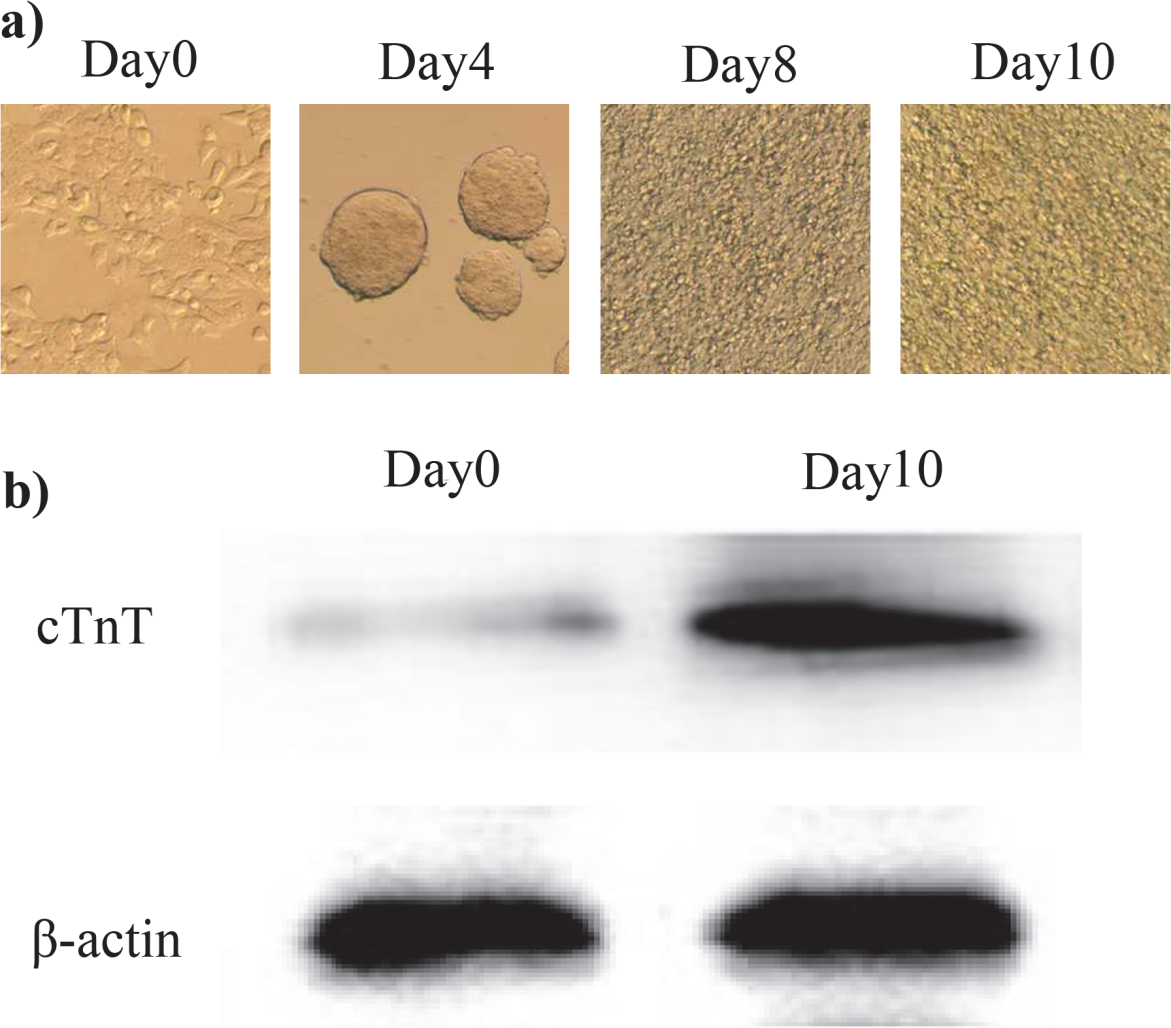
The morphological changes during P19 differentiation (×10) b) The expression of the cTnT protein during differentiation (a). To investigate the differentiation of P19 cells into mature cardiomyocytes, we used western blotting to identify the expression of the cTnT protein during differentiation. The expression of β-actin was used as a internal control.

P19 cells were differentiated into the cardiomyocytes by 1%DMSO. We used an inverted microscope to observe the morphological changes during differentiation. As shown in Fig 4A, P19 cells formed aggregates like balls in suspension from day 0 to day 4. From the fourth day, the EBs were cultivated in medium without DMSO. At day 8, a large number of fusiform cells were visible around the EBs, some of which showed a rhythmic beat. The beating cells continued to increase to day 10.

To examine the differentiation of P19 cells into mature cardiac myocytes, western blotting was used to investigate the cTnT protein level during differentiation. The expression of cTnT protein significantly increased in the differentiating P19 cells (Fig 4B).

### Effect of miR-20b on Cell Apoptosis

**Fig 5:**
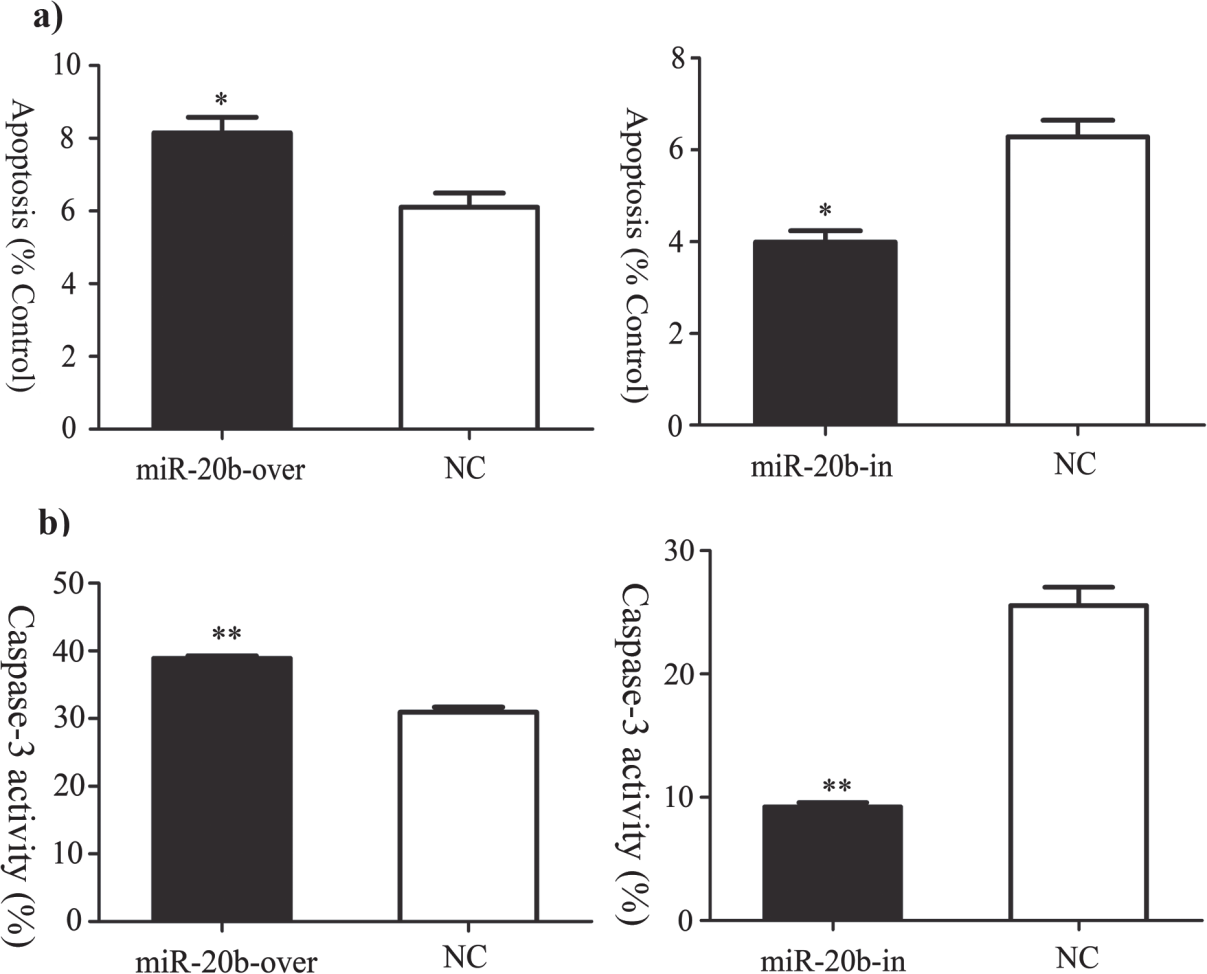
Effects of miR-20b on cell apoptosis. a) Apoptosis assayed by binding to Annexin V-APC/7-AAD. b) Apoptosis detected by measurement of Caspase-3 activity. miR-20b-over: miR-20b overexpression cells; miR-20b-in: miR-20b silenced cells. (n = 4, *: P<0.05, **: P<0.01)

The Annexin V-APC/7-AAD probe and caspase-3 activity were applied to measure cell apoptosis. Both methods indicted that miR-20b overexpression promoted apoptosis, while miR-20b silencing had the opposite effect (Fig 5).

### Effect of miR-20b on P19 Cell Differentiation

In the miR-20b overexpressing and its control groups, the beat cells both markedly increased at differentiation day 10, but with no statistical differences. In the miR-20b silenced cells, no beating cells were visible at day10, or even at day14.

**Fig 6:**
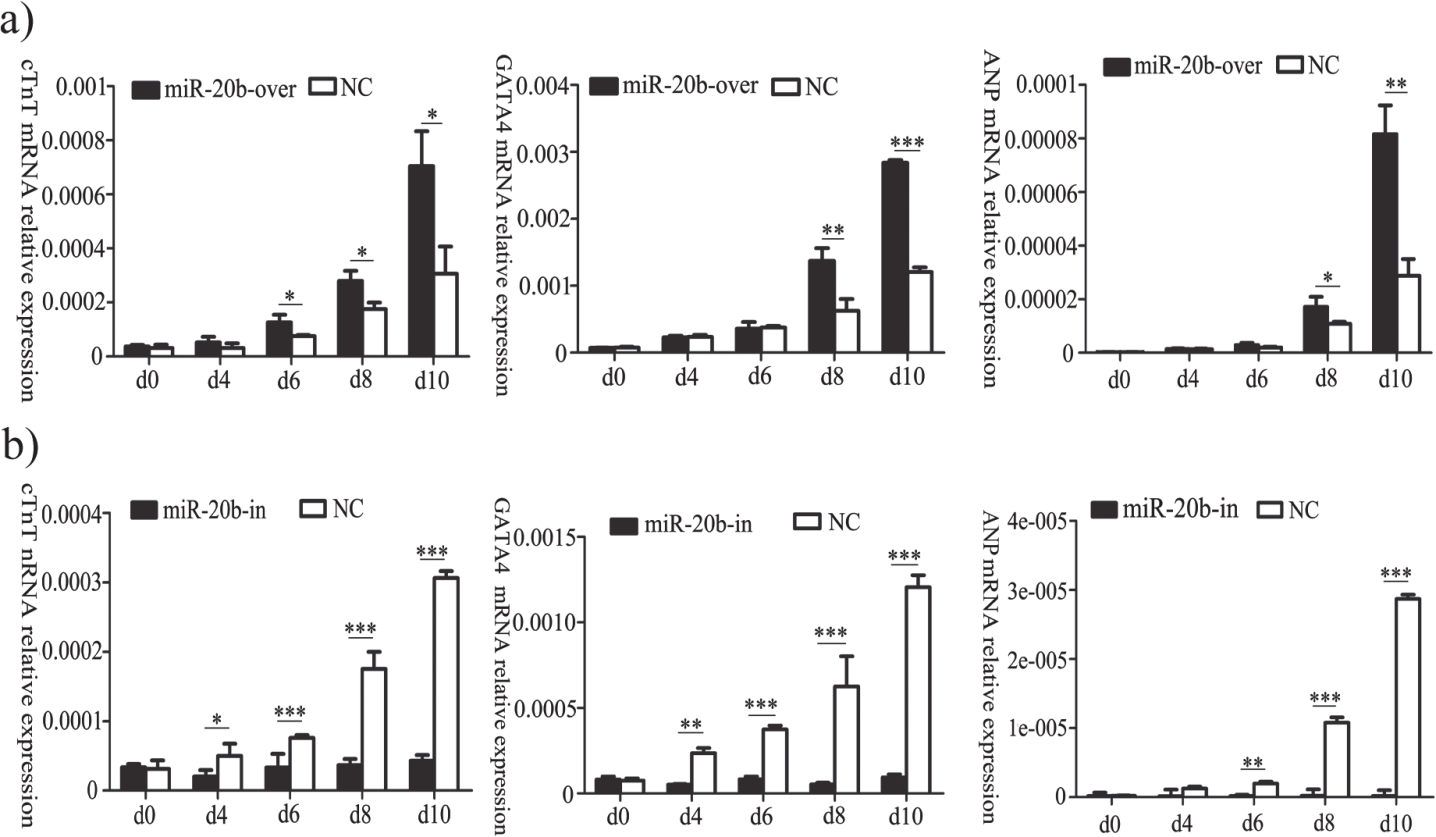
Effects of miR-20b on differentiation markers by qPCR. a): in the miR-20b overexpression cells; b): in the miR-20b silencing group. (n = 3, *: P<0.05, **: P<0.01, ***: P<0.001)

The regulation of transcription factors plays key roles during cardiomyocytes’ differentiation. Numerous studies have indicated that cTnT, cardiac myosin (α-MHC), GATA4, MEF2C, ANP and Nkx2.5 are marker genes of cardiomyocyte differentiation. We applied qPCR to identify the relative expression of cTnT, GATA4 and ANP among the miR-20b differential expression groups. The relative expression of the three genes was on the rise during differentiation in the miR-20b overexpressing cells and the controls, whereas there was no obvious change in the miR-20b silenced cells. In addition, their expressions in the miR-20b overexpressing cells were significantly higher than those in the empty vector control cells at the same differentiation time (Fig 6, *: P<0.05, **: P<0.01, ***: P<0.001). These results suggested that miR-20b overexpression promoted the differentiation of P19 cells into cardiomyocytes, whereas miR-20b silencing inhibited differentiation.

### Effect of miR-20b on the BMP Signaling Pathway

**Fig 7:**
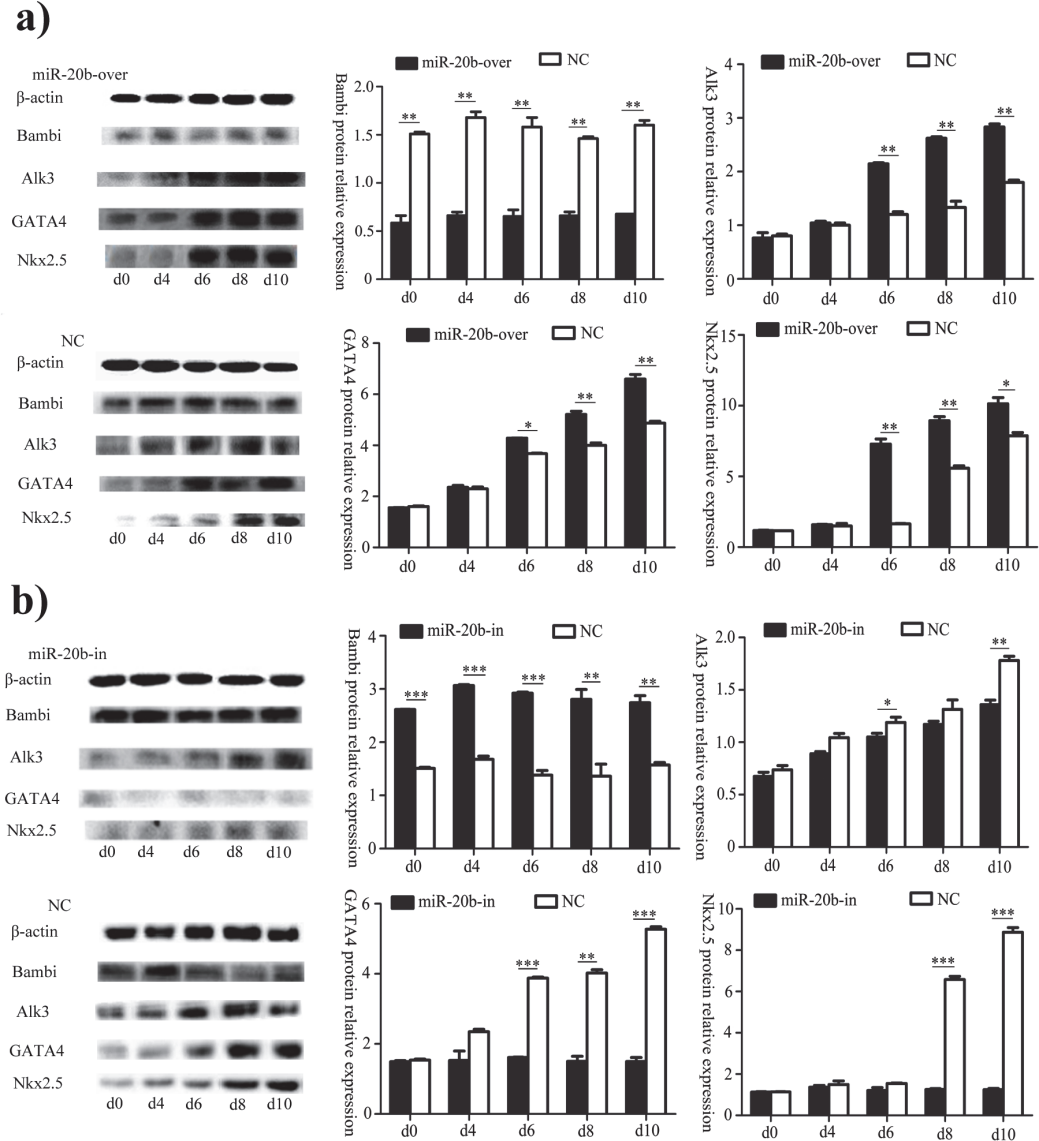
Effects of miR-20b on the BMP signaling pathway, as assessed by by western blotting. a): in the miR-20b overexpression group; b): in miR-20b silenced group. (n = 3, *: P<0.05, **: P<0.01, ***: P<0.001)

We have validated that Bambi was the direct target gene of mmu-miR-20b. Bambi inhibit the BMP signal pathway, in which BMP type I receptor ligands (BMPR1a or Alk3) functioned as a switch, and GATA4 and Nkx2.5 acted as the effector molecule. In the miR-20b overexpression group, the protein level of Bambi was much lower than in the control, and the levels of GATA4, Alk3 and Nkx2.5 were much higher, which suggested that miR-20b overexpression increased the activity of the BMP signaling pathway. Compared with the control, the protein expression of Bambi in the miR-20b silenced was significantly increased, and the levels of the other three proteins were all significantly decreased, which indicated that miR-20b silencing suppressed the activation of the BMP signaling pathway (Fig 7).

### Effect of miR-20b on Mitochondrial DNA Copy Number

**Fig 8:**
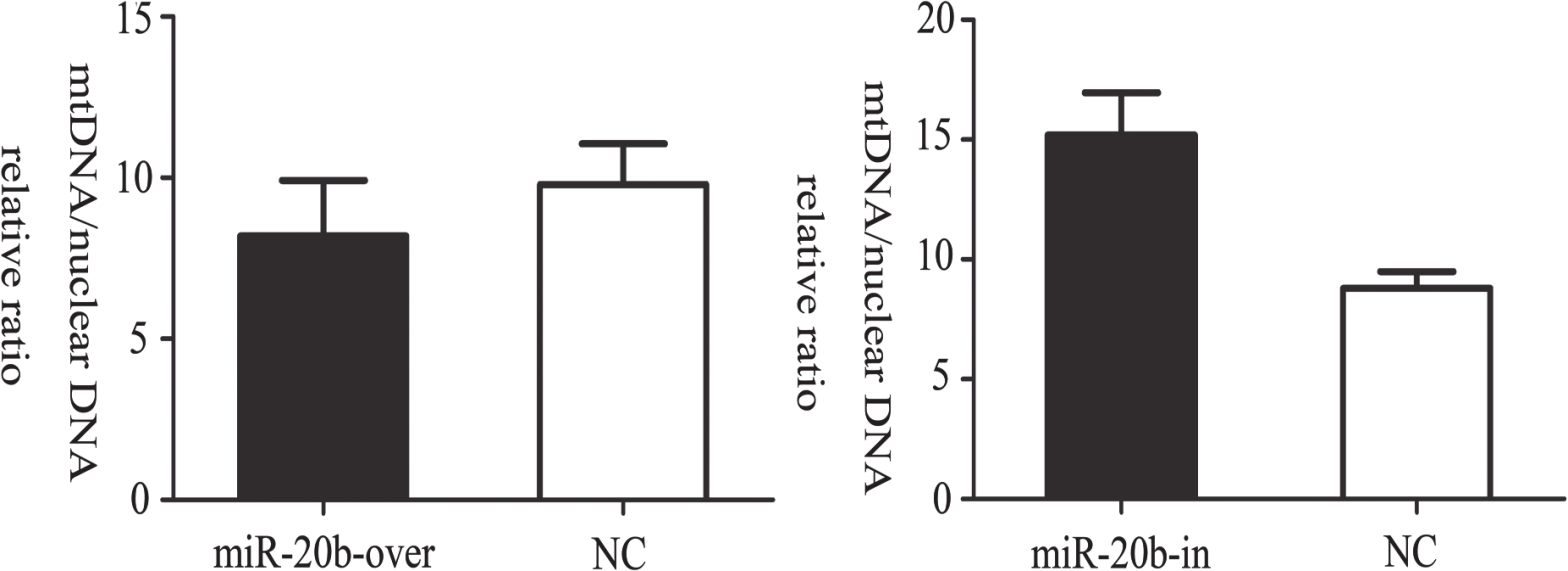
Effects of miR-20b on the mitochondrial DNA (mtDNA) copy number. On the 10th day of differentiation, cellular mtDNA content was assessed by qRT-PCR analysis with primers designed to target the *CYTB* and 28 S rRNA genes (n = 6). miR-20b-over: miR-20b overexpression cells; miR-20b-in: miR-20b silenced cells. P > 0.05 in comparison with negative control (NC) cells.

Robin et al. indicated that the mtDNA copy number per mitochondrion is generally constant in most mammalian cell types [38]. The mtDNA copy number is usually used to represent the relative quantity of mitochondria and reflects mitochondrial function. We monitored the mitochondrial and genomic DNA in miR-20b overexpressing, silenced and control cells using real-time PCR. As shown in Fig 8, there was no significant difference in mtDNA copy number among the four groups (P>0.05).

### Effect of miR-20b on Cellular ATP production

**Fig 9:**
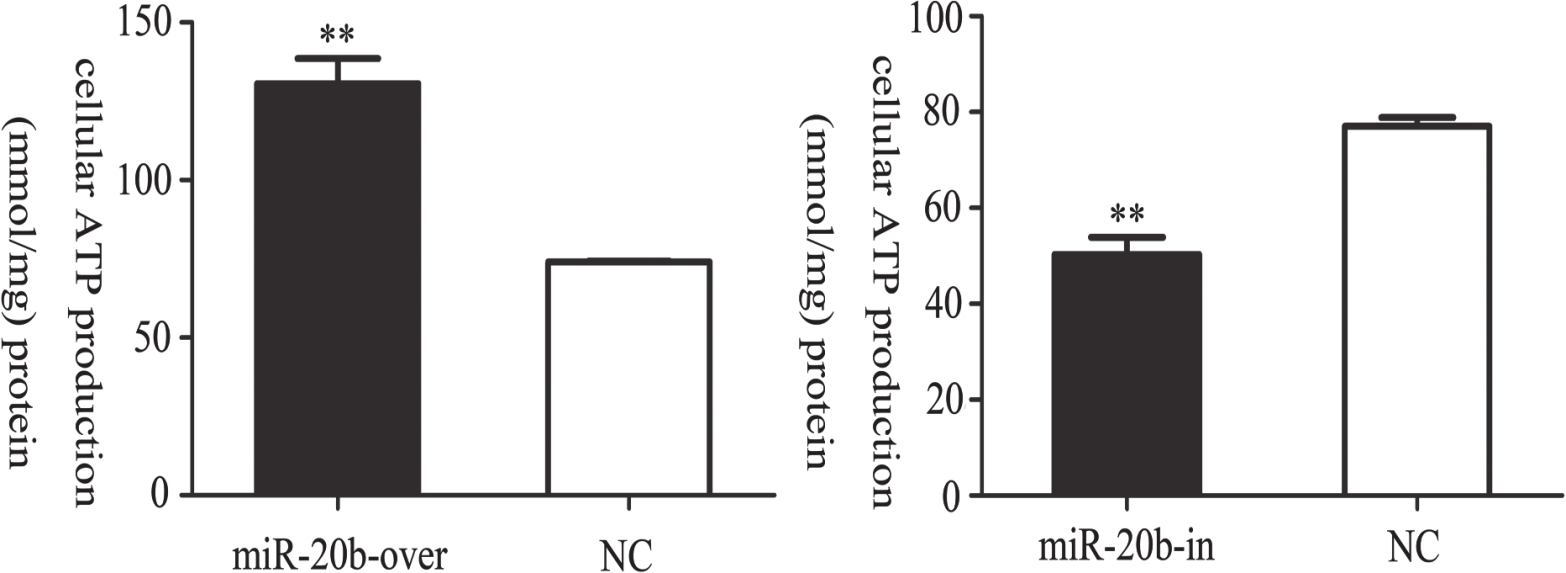
Effects of miR-20b on intracellular ATP levels. Total cellular ATP levels were measured using a luciferase-based luminescence assay kit. The results are representative of those values obtained from three independent experiments (n = 6). Values represent the mean ± standard deviation (SD). miR-20b-over: miR-20b overexpression cells; miR-20b-in: miR-20b silenced cells. (**: P< 0.01)

In cardiomyocytes, energy mainly originates from mitochondria, where ATP is generated. When the function of mitochondrial is impaired, the production of ATP decreases. Here, we found that the cellular ATP production increased in the miR-20b overexpressing cells, and decreased in the miR-20b silenced cells (Fig 9, **: P<0.01).

### Effect of miR-20b on Intracellular ROS Levels

**Fig 10:**
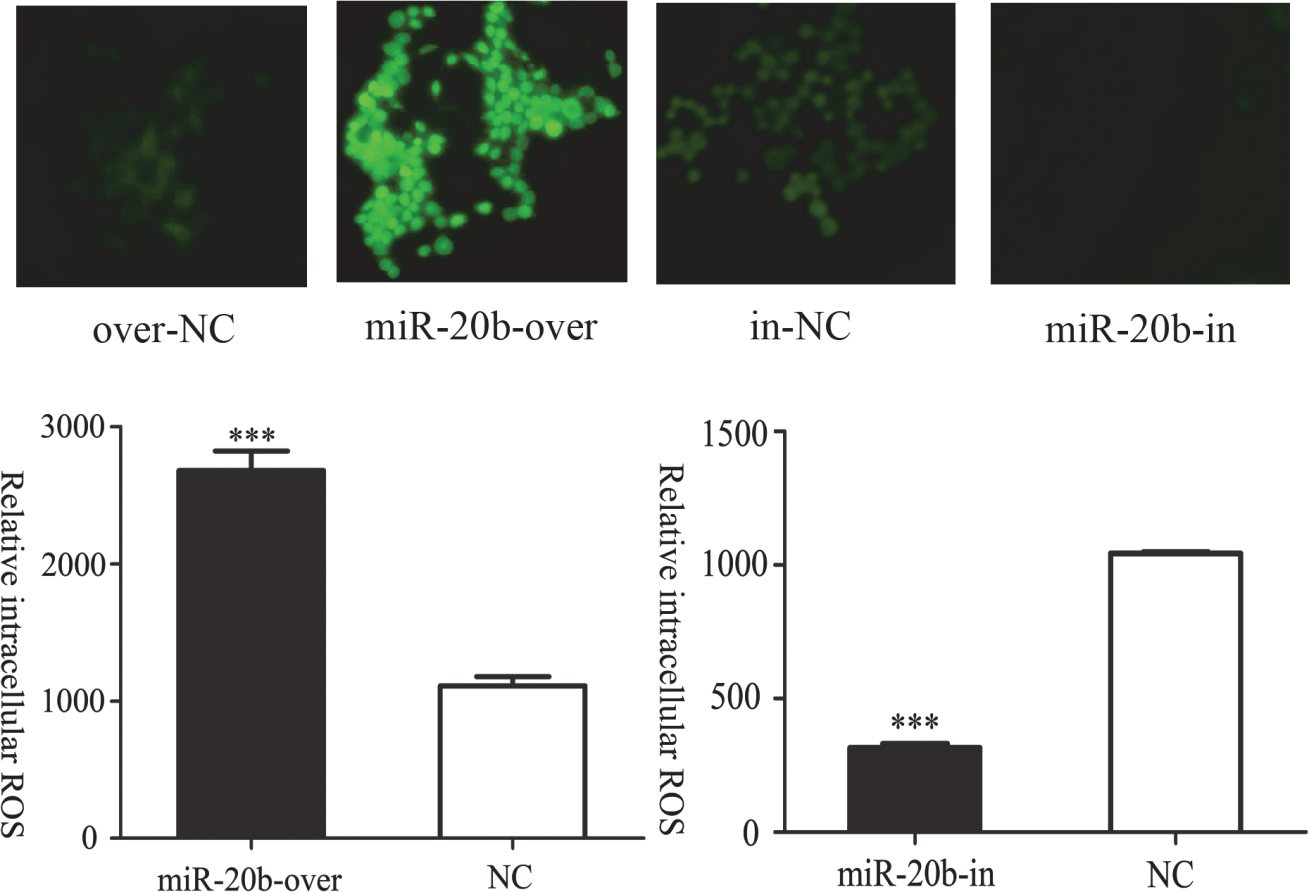
Effects of miR-20b on intracellular reactive oxygen species (ROS) content in differentiated cells. The ROS levels were measured on the 10th day of differentiation, using a FACScan flow cytometer (excitation at 488 nm, emission at 530 nm), and then viewed using a confocal laser-scanning microscope (n = 5). miR-20b-over: miR-20b overexpression cells; miR-20b-in: miR-20b silenced cells. (***: P<0.001)

The DCFDA probe could be oxidized into DCF, which has high fluorescence intensity. Hence, detecting the intensity of DCF monitored the ROS levels, which in turn reflect the mitochondrial function. The fluorescence signals in miR-20b overexpressing cells were higher than those of the control, indicating that miR-20b overexpression increased ROS generation; silencing miR-20b had the opposite effect (Fig 10, ***: P<0.001).

### Effect of miR-20b on the MMP

**Fig 11:**
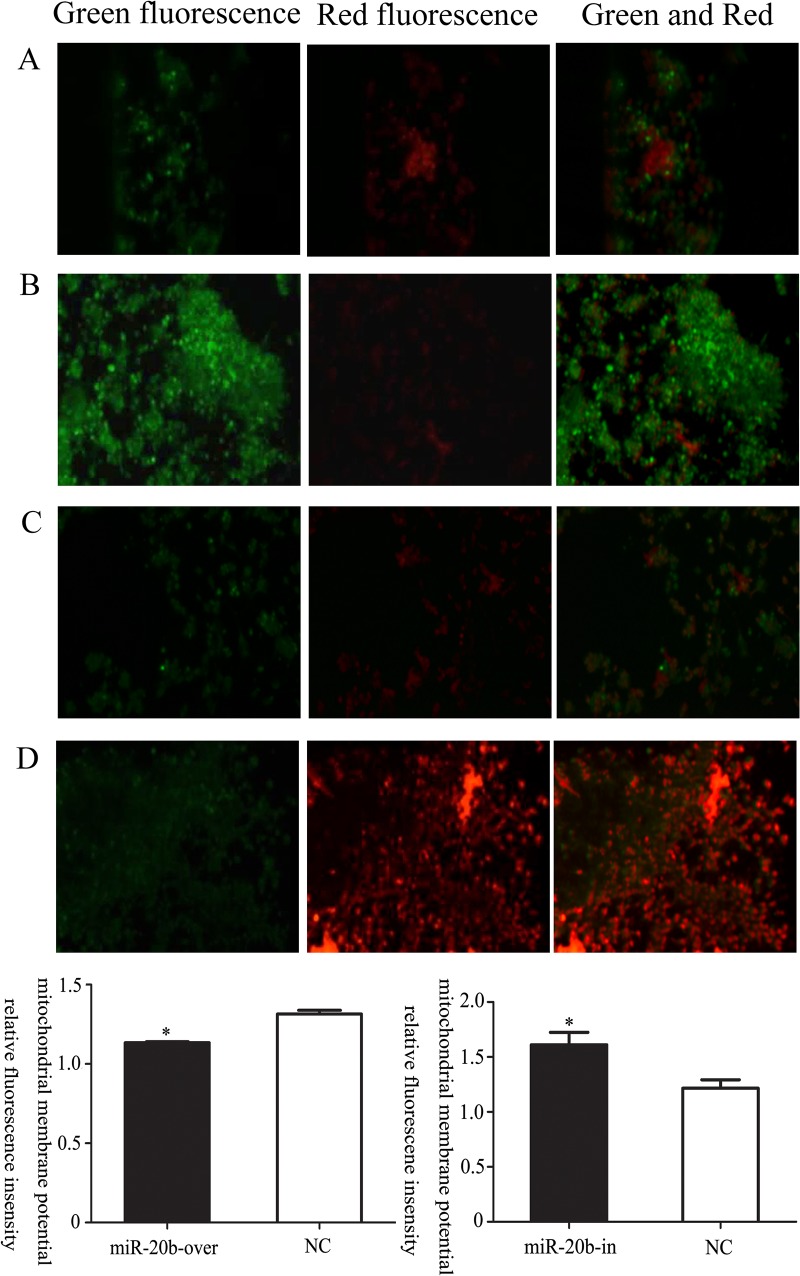
Effects of miR-20b on mitochondrial membrane potential (MMP) in differentiated cells. A: control for miR-20b overexpression; B: miR-20b overexpression; C: control for miR-20b silencing; D: miR-20b silencing. (*: P<0.05)

JC-1 is a fluorescent probe that applied widely to detect the MMP. When the MMP is high, JC-1 aggregates into a polymer to generate a red fluorescence in the mitochondrial matrix. When the MMP is low, JC-1 produces a green fluorescence without aggregating. In this study, the relative proportion of red and green fluorescence under fluorescence microscopy and flow cytometry was used to assess MMP. Both methods showed that miR-20b overexpression produced a decrease in the MMP, while miR-20b silencing resulted in an increase (Fig 11, *: P<0.05).

## Discussion

CHDs are the most common major congenital malformation and also the leading cause of infant morbidity and mortality. CHDs comprise numerous structural and functional abnormalities of the heart and great vessels [39, 40]. In humans, 60% of protein-encoding genes were regulated by miRNAs [41]. Although many miRNAs are correlated with cardiogenesis and regarded as new biomarkers and therapeutic targets [42], their specific roles remain unclear.

In the present study, we constructed a stable P19 mmu-miR-20b overexpression line and a stable miR-20b silenced cell line. MiR-20b silencing cells were achieved by using “MiRNA sponge”, containing some antisense sequences complementary to miRNA [43,44]. Essentially, miRNA sponge is mRNA. The presence of several binding sites in the 3′UTR, miRNA sponges can efficiently and persistently inhibit miRNA endogenous expression. More sites, more efficiently.

Their ability to differentiate into cardiomyocytes has led to P19 cells being used widely in studies of pathological and physiological processes of cardiogenesis. GATA4, cTnT and ANP are often applied frequently to identify the formation of cardiac cells [45,46]. The relative expressions of the three genes increased during P19 differentiation, and were significantly higher in the miR-20b overexpression group, while there was no difference in the miR-20b silenced cells. This suggested that miR-20b overexpression promoted differentiation, while miR-20b silencing inhibited it. The BMP signaling pathway plays an important role in cardiac differentiation. BMPs promotes the phosphorylation of downstream genes by binding to their receptors, thereby influencing the expression of key cardiac genes, e.g. GATA4, Nkx2.5. BMP-2 knockout mice showed embryonic amniotic malformations and cardiac hypoplasia [47]. The mesoderm was affected in BMP-4 knockout mice [48]. Moreover, Alk3 knockout mice died of endocardial cushion, trabecular hypoplasia and ventricular septal defect in mid-embryonic development [49]. Nkx2.5, a homologous gene of the NK-2 class, is mainly expressed in heart tissue and cardiac precursor cells. Cardiogenesis broke down in the absence of Nkx2.5 [50]. GATA4 belongs to the cardiogenic GATA subline and plays a key role in the origin and development of heart, like Nkx2.5. In the previous study, overexpression of BMP-2 in the non-cardiac-derived embryo layer of chickens induced ectopic expression of GATA-4 and Nkx2.5 to promote heart development [51]. Numbers of studies have demonstrated that BMP and activin membrane-bound inhibitor (Bambi), a pseudo-receptor of BMPs, affects the activity of the BMP signaling pathway by interrupting normal binding to suppress cardiac differentiation [29,30]. We showed that miR-20b inhibited the protein expression of Bambi by acting directly on the 3′UTR of its mRNA. On the basis of the results, we hypothesized that more active BMPs were released to bind with BMP receptors because of the suppression of Bambi by miR-20b overexpression. Subsequently, the more active BMP signaling pathway increased the protein expression of Nkx2.5 and GATA4, leading to promotion of cardiac differentiation. By contrast, miR-20b silencing inhibited differentiation by reducing the activity of the BMP signaling pathway.

Flow cytometry and measurement of caspase-3 activity indicated that apoptosis of P19 cells with overexpressing miR-20b was significantly upregulated, whereas silencing significantly inhibited apoptosis. The molecular mechanism that underlay this effect remained unknown. Some scientists believed that mitochondrial function influenced transmission and amplification of apoptosis signals [52]. It was also suggested that cell apoptosis induced dynamic changes in the function and structure of mitochondria [53,54]. Therefore, in the present study, we further explored the effects of miR-20b on mitochondrial function in P19 cells.

MtDNA copy number was generally used to represent the number of mitochondria, as an indicator reflecting the mitochondrial function [55]. Neither overexpression nor silencing of miR-20b caused a significant change in mtDNA copy number. In general, the fragile mtDNA was more vulnerable to damage than nuclear DNA [56]. In addition, 90% of ROS were generated from mitochondria. When cell apoptosis increased, mitochondria became dysfunctional and ROS accumulated as an intermediate [57]. Meanwhile, accumulation of ROS promoted mitochondrial dysfunction [58]. It seemed like positive feedback. We used DCFDA probe to demonstrate that miR-20b overexpression increased the ROS level of cardiomyocytes, suggesting that miR-20b overexpression might damage mitochondria whereas the silencing had an opposite effect. In theory, the deterioration of mitochondrial function caused by increased ROS would destroy the integrity of the mtDNA, leading to a reduction of the mtDNA copy number. However, there was no significant difference in mtDNA copy number in fact. We hypothesized that there were other compensatory mechanisms that protected impaired mitochondria.

The energy of myocardial cells is generated mainly from mitochondrial ATP [59]. When mitochondria are impaired, the decline in ATP synthesis would lead to an inadequate supply of cardiomyocyte energy, possibly inducing stagnation of cardiogenesis. In theory, a lower cellular ATP production would be accompanied by an impaired in mitochondrial function. However, in miR-20b overexpressing cells, the ATP production was increased when the increased ROS promoted mitochondrial dysfunction. This also suggested the presence of as-yet-unknown compensatory mechanisms.

The MMP is defined as an electrochemical gradient of protons between the inner and outer membranes, which can directly reflect mitochondrial function. When mitochondria are impaired, the varying permeability of the membranes leads to a decrease in the MMP. In the present study, the MMP in miR-20b overexpressing cells was reduced, suggesting that the mitochondria were damaged. MMP in the silenced was increased, which might be helpful to maintain normal mitochondrial function. The decreased MMP could cause mitochondria to swell, changing their permeability. Cytochrome c would then move into the cytoplasm and some protease systems would be activated, resulting in apoptosis increased [60]. Thus, we concluded that the increased apoptosis in miR-20b overexpressing cells was likely to be associated with the reduced MMP and impaired mitochondria.

In summary, we demonstrated that miR-20b overexpression increased apoptosis and promoted differentiation in P19 cells by promoting the activation of the BMP signaling pathway. In addition, miR-20b overexpression induced mitochondrial impairment in P19 cells during differentiation, which was characterized by lower MMP, raised ATP synthesis and increased ROS levels. The effects of miR-20b silencing were the exact opposite to those of overexpression. These findings may provide a new viewpoint into the mechanisms of cardiac abnormalities. Furthermore, miR-20b may be a potential new therapeutic target for CHDs. Therefore, the next step is to confirm whether abnormalities in miR-20b expression contribute to CHD in vivo.

## References

[1] Zaffran S, Frasch M. Early signals in cardiac development. Circ Res 2002;91(6):457–69. 12242263 10.1161/01.res.0000034152.74523.a8

[2] Miyazono K, Kamiya Y, Morikawa M. Bone morphogenetic protein receptors and signal transduction. J Biochem 2010;147(1):35–51. 10.1093/jb/mvp148 19762341

[3] Bajolle F, Zaffran S, Bonnet D. Genetics and embryological mechanisms of congenital heart diseases. Arch Cardiovasc Dis 2009;102(1):59–63. 10.1016/j.acvd.2008.06.020 19233110

[4] Zhao Y, Ransom JF, Li A, Vedantham V, von Drehle M, Muth AN, et al Dysregulation of cardiogenesis, cardiac conduction, and cell cycle in mice lacking miRNA-1-2. Cell 2007;129(2):303–17. 17397913 10.1016/j.cell.2007.03.030

[5] Morton SU, Scherz PJ, Cordes KR, Ivey KN, Stainier DY, Srivastava D. microRNA-138 modulates cardiac patterning during embryonic development. Proc Natl Acad Sci U S A 2008;105(46):17830–5. 10.1073/pnas.0804673105 19004786 PMC2582580

[6] Qin DN, Qian L, Hu DL, Yu ZB, Han SP, Zhu C, et al Effects of miR-19b overexpression on proliferation, differentiation, apoptosis and Wnt/beta-catenin signaling pathway in P19 cell model of cardiac differentiation in vitro. Cell Biochem Biophys 2013;66(3):709–22. 10.1007/s12013-013-9516-9 23443808

[7] Meberg A, Lindberg H, Thaulow E. Congenital heart defects: the patients who die. Acta Paediatr 2005;94(8):1060–5. 16188850 10.1111/j.1651-2227.2005.tb02046.x

[8] Trojnarska O, Grajek S, Katarzynski S, Kramer L. Predictors of mortality in adult patients with congenital heart disease. Cardiol J 2009;16(4):341–7. 19653177

[9] Bruneau BG. The developmental genetics of congenital heart disease. Nature 2008;451(7181):943–8. 10.1038/nature06801 18288184

[10] Olson EN. Gene regulatory networks in the evolution and development of the heart. Science 2006;313(5795):1922–7. 17008524 10.1126/science.1132292 PMC4459601

[11] Bartel DP. MicroRNAs: genomics, biogenesis, mechanism, and function. Cell 2004;116(2):281–97. 14744438 10.1016/s0092-8674(04)00045-5

[12] Ambros V. The functions of animal microRNAs. Nature 2004;431(7006):350–5. 15372042 10.1038/nature02871

[13] Kloosterman WP, Plasterk RH. The diverse functions of microRNAs in animal development and disease. Dev Cell 2006;11(4):441–50. 17011485 10.1016/j.devcel.2006.09.009

[14] Garzon R, Pichiorri F, Palumbo T, Visentini M, Aqeilan R, Cimmino A, et al MicroRNA gene expression during retinoic acid-induced differentiation of human acute promyelocytic leukemia. Oncogene 2007;26(28):4148–57. 17260024 10.1038/sj.onc.1210186

[15] Mack GS. MicroRNA gets down to business. Nat Biotechnol 2007;25(6):631–8. 17557095 10.1038/nbt0607-631

[16] Thum T, Catalucci D, Bauersachs J. MicroRNAs: novel regulators in cardiac development and disease. Cardiovasc Res 2008;79(4):562–70. 10.1093/cvr/cvn137 18511432

[17] Catalucci D, Latronico MV, Condorelli G. MicroRNAs control gene expression: importance for cardiac development and pathophysiology. Ann N Y Acad Sci 2008;1123:20–9. 10.1196/annals.1420.004 18375574

[18] Harada M, Luo X, Murohara T, Yang B, Dobrev D, Nattel S. MicroRNA regulation and cardiac calcium signaling: role in cardiac disease and therapeutic potential. Circ Res 2014;114(4):689–705. 10.1161/CIRCRESAHA.114.301798 24526675

[19] Nabialek E, Wanha W, Kula D, Jadczyk T, Krajewska M, Kowalowka A, et al Circulating microRNAs (miR-423-5p, miR-208a and miR-1) in acute myocardial infarction and stable coronary heart disease. Minerva Cardioangiol 2013;61(6):627–37. 24253456

[20] Chen PY, Manninga H, Slanchev K, Chien M, Russo JJ, Ju J, et al The developmental miRNA profiles of zebrafish as determined by small RNA cloning. Genes Dev 2005;19(11):1288–93. 15937218 10.1101/gad.1310605 PMC1142552

[21] Ahn HW, Morin RD, Zhao H, Harris RA, Coarfa C, Chen ZJ, et al MicroRNA transcriptome in the newborn mouse ovaries determined by massive parallel sequencing. Mol Hum Reprod 2010;16(7):463–71. 10.1093/molehr/gaq017 20215419 PMC2882868

[22] Zhu J, Chen L, Zou L, Yang P, Wu R, Mao Y, et al MiR-20b, -21, and -130b inhibit PTEN expression resulting in B7-H1 over-expression in advanced colorectal cancer. Hum Immunol 2014;75(4):348–53. 10.1016/j.humimm.2014.01.006 24468585

[23] Cascio S, D'Andrea A, Ferla R, Surmacz E, Gulotta E, Amodeo V, et al miR-20b modulates VEGF expression by targeting HIF-1 alpha and STAT3 in MCF-7 breast cancer cells. J Cell Physiol 2010;224(1):242–9. 10.1002/jcp.22126 20232316

[24] Li Jian-Yi, Zhang Yang, Zhang Wen-Hai, Li Jian-Yi, Zhang Yang, Zhang Wen-Hai, et al Differential Distribution of miR-20a and miR-20b may Underly Metastatic Heterogeneity of Breast Cancers. Asian Pacific Journal of Cancer Prevention 2012;13:1901–6. 22901144 10.7314/apjcp.2012.13.5.1901

[25] Callis TE, Cao D, Wang DZ. Bone morphogenetic protein signaling modulates myocardin transactivation of cardiac genes. Circ Res 2005;97(10):992–1000. 16224060 10.1161/01.RES.0000190670.92879.7d PMC2930260

[26] Monzen K, Shiojima I, Hiroi Y, Kudoh S, Oka T, Takimoto E, et al Bone morphogenetic proteins induce cardiomyocyte differentiation through the mitogen-activated protein kinase kinase kinase TAK1 and cardiac transcription factors Csx/Nkx-2.5 and GATA-4. Mol Cell Biol 1999;19(10):7096–105. 10490646 10.1128/mcb.19.10.7096 PMC84704

[27] Wang J, Greene SB, Martin JF. BMP signaling in congenital heart disease: new developments and future directions. Birth Defects Res A Clin Mol Teratol 2011;91(6):441–8. 10.1002/bdra.20785 21384533 PMC3124406

[28] de Pater E, Ciampricotti M, Priller F, Veerkamp J, Strate I, Smith K, et al Bmp signaling exerts opposite effects on cardiac differentiation. Circ Res 2012;110(4):578–87. 10.1161/CIRCRESAHA.111.261172 22247485 PMC4924880

[29] Gazzerro E, Canalis E. Bone morphogenetic proteins and their antagonists. Rev Endocr Metab Disord 2006;7(1–2):51–65. 17029022 10.1007/s11154-006-9000-6

[30] Chen J, Bush JO, Ovitt CE, Lan Y, Jiang R. The TGF-beta pseudoreceptor gene Bambi is dispensable for mouse embryonic development and postnatal survival. Genesis 2007;45(8):482–6. 17661381 10.1002/dvg.20320 PMC2376806

[31] Onichtchouk D, Chen YG, Dosch R, Gawantka V, Delius H, Massague J, et al Silencing of TGF-beta signalling by the pseudoreceptor BAMBI. Nature 1999;401(6752):480–5. 10519551 10.1038/46794

[32] van der Heyden MA, Defize LH. Twenty one years of P19 cells: what an embryonal carcinoma cell line taught us about cardiomyocyte differentiation. Cardiovasc Res 2003;58(2):292–302. 12757864 10.1016/s0008-6363(02)00771-x

[33] van der Heyden MA, van Kempen MJ, Tsuji Y, Rook MB, Jongsma HJ, Opthof T. P19 embryonal carcinoma cells: a suitable model system for cardiac electrophysiological differentiation at the molecular and functional level. Cardiovasc Res 2003;58(2):410–22. 12757875 10.1016/s0008-6363(03)00247-5

[34] Wen J, Xia Q, Lu C, Yin L, Hu J, Gong Y, et al Proteomic analysis of cardiomyocytes differentiation in mouse embryonic carcinoma P19CL6 cells. J Cell Biochem 2007;102(1):149–60. 17520663 10.1002/jcb.21285

[35] Kaaman M, Sparks LM, van Harmelen V, Smith SR, Sjolin E, Dahlman I, et al Strong association between mitochondrial DNA copy number and lipogenesis in human white adipose tissue. Diabetologia 2007;50(12):2526–33. 17879081 10.1007/s00125-007-0818-6

[36] Shen YH, Song GX, Liu YQ, Sun W, Zhou LJ, Liu HL, et al Silencing of FABP3 promotes apoptosis and induces mitochondrion impairment in embryonic carcinoma cells. J Bioenerg Biomembr 2012;44(3):317–23. 10.1007/s10863-012-9439-y 22528395

[37] Maxwell DP, Wang Y, McIntosh L. The alternative oxidase lowers mitochondrial reactive oxygen production in plant cells. Proc Natl Acad Sci U S A 1999;96(14):8271–6. 10393984 10.1073/pnas.96.14.8271 PMC22224

[38] Robin ED, Wong R. Mitochondrial DNA molecules and virtual number of mitochondria per cell in mammalian cells. J Cell Physiol 1988;136(3):507–13. 3170646 10.1002/jcp.1041360316

[39] Allan L. Antenatal diagnosis of heart disease. Heart 2000;83(3):367 10677423 10.1136/heart.83.3.367 PMC1729333

[40] Eleftheriades M, Tsapakis E, Sotiriadis A, Manolakos E, Hassiakos D, Botsis D. Detection of congenital heart defects throughout pregnancy; impact of first trimester ultrasound screening for cardiac abnormalities. J Matern Fetal Neonatal Med 2012;25(12):2546–50. 10.3109/14767058.2012.703716 22712625

[41] Friedman RC, Farh KK, Burge CB, Bartel DP. Most mammalian mRNAs are conserved targets of microRNAs. Genome Res 2009;19(1):92–105. 10.1101/gr.082701.108 18955434 PMC2612969

[42] Zhu S, Cao L, Zhu J, Kong L, Jin J, Qian L, et al Identification of maternal serum microRNAs as novel non-invasive biomarkers for prenatal detection of fetal congenital heart defects. Clin Chim Acta 2013;424:66–72. 10.1016/j.cca.2013.05.010 23707860

[43] Ebert MS, Sharp PA. MicroRNA sponges: progress and possibilities. RNA 2010;16(11):2043–50. 10.1261/rna.2414110 20855538 PMC2957044

[44] Ebert MS, Neilson JR, Sharp PA. MicroRNA sponges: competitive inhibitors of small RNAs in mammalian cells. Nat Methods 2007;4(9):721–6. 17694064 10.1038/nmeth1079 PMC3857099

[45] Ku PM, Chen LJ, Liang JR, Cheng KC, Li YX, Cheng JT. Molecular role of GATA binding protein 4 (GATA-4) in hyperglycemia-induced reduction of cardiac contractility. Cardiovasc Diabetol 2011;10:57 10.1186/1475-2840-10-57 21702924 PMC3141394

[46] Kehat I, Gepstein A, Spira A, Itskovitz-Eldor J, Gepstein L. High-resolution electrophysiological assessment of human embryonic stem cell-derived cardiomyocytes: a novel in vitro model for the study of conduction. Circ Res 2002;91(8):659–61. 12386141 10.1161/01.res.0000039084.30342.9b

[47] Zhang H, Bradley A. Mice deficient for BMP2 are nonviable and have defects in amnion/chorion and cardiac development. Development 1996;122(10):2977–86. 8898212 10.1242/dev.122.10.2977

[48] Winnier G, Blessing M, Labosky PA, Hogan BL. Bone morphogenetic protein-4 is required for mesoderm formation and patterning in the mouse. Genes Dev 1995;9(17):2105–16. 7657163 10.1101/gad.9.17.2105

[49] Mishina Y, Suzuki A, Ueno N, Behringer RR. Bmpr encodes a type I bone morphogenetic protein receptor that is essential for gastrulation during mouse embryogenesis. Genes Dev 1995;9(24):3027–37. 8543149 10.1101/gad.9.24.3027

[50] Lyons I, Parsons LM, Hartley L, Li R, Andrews JE, Robb L, et al Myogenic and morphogenetic defects in the heart tubes of murine embryos lacking the homeo box gene Nkx2-5. Genes Dev 1995;9(13):1654–66. 7628699 10.1101/gad.9.13.1654

[51] Schultheiss TM, Burch JB, Lassar AB. A role for bone morphogenetic proteins in the induction of cardiac myogenesis. Genes Dev 1997;11(4):451–62. 9042859 10.1101/gad.11.4.451

[52] Crow MT, Mani K, Nam YJ, Kitsis RN. The mitochondrial death pathway and cardiac myocyte apoptosis. Circ Res 2004;95(10):957–70. 15539639 10.1161/01.RES.0000148632.35500.d9

[53] Martinou JC, Youle RJ. Mitochondria in apoptosis: Bcl-2 family members and mitochondrial dynamics. Dev Cell 2011;21(1):92–101. 10.1016/j.devcel.2011.06.017 21763611 PMC3156409

[54] Ugarte-Uribe B, Garcia-Saez AJ. Membranes in motion: mitochondrial dynamics and their role in apoptosis. Biol Chem 2014;395(3):297–311. 10.1515/hsz-2013-0234 24184992

[55] Shi CM, Xu GF, Yang L, Fu ZY, Chen L, Fu HL, et al Overexpression of TFAM protects 3T3-L1 adipocytes from NYGGF4 (PID1) overexpression-induced insulin resistance and mitochondrial dysfunction. Cell Biochem Biophys 2013;66(3):489–97. 10.1007/s12013-012-9496-1 23274913

[56] Kang D, Hamasaki N. Maintenance of mitochondrial DNA integrity: repair and degradation. Curr Genet 2002;41(5):311–22. 12185497 10.1007/s00294-002-0312-0

[57] Matsusaka T, Ichikawa I. Biological functions of angiotensin and its receptors. Annu Rev Physiol 1997;59:395–412. 9074770 10.1146/annurev.physiol.59.1.395

[58] Lesnefsky EJ, Hoppel CL. Cardiolipin as an oxidative target in cardiac mitochondria in the aged rat. Biochim Biophys Acta 2008;1777(7–8):1020–7. 10.1016/j.bbabio.2008.09.005 18515061 PMC2527751

[59] Hatefi Y. The mitochondrial electron transport and oxidative phosphorylation system. Annu Rev Biochem 1985;54:1015–69. 2862839 10.1146/annurev.bi.54.070185.005055

[60] Marre M, Bernadet P, Gallois Y, Savagner F, Guyene TT, Hallab M, et al Relationships between angiotensin I converting enzyme gene polymorphism, plasma levels, and diabetic retinal and renal complications. Diabetes 1994;43(3):384–8. 8314010 10.2337/diab.43.3.384

